# Remembering Keisuke Fujita, MD, PhD, President and Founder of Fujita Health University

**DOI:** 10.20407/fmj.2021-001

**Published:** 2021-03-20

**Authors:** Shosuke Ito

**Affiliations:** Institute for Melanin Chemistry, Fujita Health University, Toyoake, Aichi, Japan

## Dr. Keisuke Fujita as Administrator and Educator

It has been just a quarter of a century since Dr. Keisuke Fujita passed away. Keisuke Fujita, MD, PhD (1925–1995) founded Fujita Gakuen (“academy”) in 1964, Fujita Health University (FHU) College in 1966, FHU School of Health Sciences in 1968, and FHU School of Medicine in 1972. He also established the Institute of Comprehensive Medical Science in 1972. Dr. Fujita was an energetic and tireless administrator. I was fortunate to work under his supervision at the Center for Pharmacognosy (Shoyaku Kenkyu Juku) in 1977 to study the biochemical properties of aloe carboxypeptidase.^1^ I saw there how as the head of Fujita Gakuen, he worked beyond midnight almost every day and did so seven days a week. I vividly recall that he often said, “Eat well and work hard.”

Dr. Fujita was also a devoted educator. He certainly followed his own saying: he worked hard to establish a comprehensive educational system such that all Fujita Gakuen students were able to pass national examinations to obtain licenses in various fields of the medical sciences. One of his achievements in this regard was that he edited *Medical Technology Nyumonsho* (“essence”) based on the fact that questions in national examinations were mostly (over 80%) prepared based on past questions. The *Nyumonsho* consisted of minimal information to solve all of the questions presented in the past national examinations. I was fortunate that I helped him edit the first edition of *Medical Technology Nyumonsho* while I was at the Center for Pharmacognosy.

## Dr. Keisuke Fujita as Researcher

I read with great interest “Remembering Keisuke Fujita, MD, PhD, President and Founder of Fujita Health University, and his contributions to medical science and education.” That memoir^2^ was written by Dr. Toshiharu Nagatsu, Professor Emeritus, Fujita Health University, who closely collaborated with Dr. Fujita from 1965 until Dr. Fujita’s sudden death in 1995. Dr. Nagatsu is the best person to write such a memoir. The memoir vividly and thoroughly summarizes the achievements and contributions Dr. Fujita made to medical science. It includes a list of Dr. Fujita’s 111 selected publications as well as some memorable photographs of him. People who knew Dr. Fujita personally will be able to relive their memories of him not only as an outstanding scientist but also as a devoted administrator and educator. With that memoir, younger staff members and students will be able to learn how much Dr. Fujita was devoted to medical science and education. Through his devotion and the continuation of his philosophy (Table 1 in the memoir), FHU now stands as one of the finest medical institutions in the world.

To that memoir, I wish to add here some of my personal memories of Dr. Fujita. He began his career as a scientist by publishing three papers in *Nature* in 1955 and 1958 on the experimental inhibition of cancer. On the basis of his experience, Dr. Fujita often said, “Let’s aim at publishing in the best journals in the field.” When I moved to the main campus at Toyoake in 1978, Dr. Fujita suggested that I study the anti-melanoma effect of melanin precursors based on a paper by Wick in *Nature*.^3^ Dr. Fujita, I, and colleagues were fortunate to find that one melanin precursor, 5-*S*-cysteinyldopa, exhibited high growth-inhibitory effects on melanoma cells and tumors. Encouraged by our positive results, we submitted a manuscript to *Nature* and *Science*. Our paper was not accepted by those journals; however, it was published in *Cancer Research*,^4^ which is the best journal in its field. Dr. Fujita also encouraged us young researchers to establish our own lifework. I decided to make mine melanin chemistry. In that field, one of my early achievements was developing microanalytical methods to analyze eumelanin and pheomelanin (two major types of melanin pigments) in tissue samples. That study was published jointly with Dr. Fujita in *Analytical Biochemistry*.^5^ I am proud to note that among my original papers, that article has the highest citation number (433). In that regard, Dr. Nagatsu appropriately summarizes in Table 1 of his memoir the philosophy of Dr. Fujita in science: “Freedom in the selection of the research theme depending on the researcher’s interest.” Dr. Keisuke Fujita was a supportive, open-minded scientist, and he had excellent foresight of the future.
